# Supplementary material for: Motor processivity and speed determine structure and dynamics of microtubule-motor assemblies
Source: eLife. 2023 Feb 8;12:e79402. doi: 10.7554/eLife.79402 (PMC10014072; doi:10.7554/eLife.79402)
Supplement: Supplementary file 1. [file elife-79402-supp1.docx]

| **Plasmids used** |
| --- |
| pBiex1:Kif11(1-513)-(GSG)_4_-mCherry-(GSG)_2_-iLid |
| pBiex1:Kif11(1-513)-(GSG)_4_-mCherry-(GSG)_2_-micro |
| pBiex1:Kif11(1-513)-(GSG)_4_-mVenus-(GSG)_2_-iLid |
| pBiex1:K401-(GSG)_4_-mCherry-(GSG)_2_-iLid |
| pBiex1:K401-(GSG)_4_-mVenus-(GSG)_2_-micro |
| pBiex1:mVenus-(GSG)_4_-iLid-(GSG)_2_-Ncd236 |
| pBiex1:mVenus-(GSG)_4_-micro-(GSG)_2_-Ncd236 |
