# Supplementary material for: Motor processivity and speed determine structure and dynamics of microtubule-motor assemblies
Source: eLife. 2023 Feb 8;12:e79402. doi: 10.7554/eLife.79402 (PMC10014072; doi:10.7554/eLife.79402)
Supplement: Supplementary file 2. — Step size of ≈10 nm corresponding to the length of a tubulin dimer was used for estimating the motor processivities in μm units. For Ncd motors, the upper limit in processivity corresponds to that of oligomeric motor assemblies. Estimates for the decay length scales λ were made based on the motor profiles in Figure 2—figure supplement 1. [file elife-79402-supp2.docx]

| **Motor** | **Processivity (steps)** | **Processivity,**  **λ_ν_ (μm)** | **Decay length scale, λ (μm)** | **λ/λ_ν_** |
| --- | --- | --- | --- | --- |
| K401 | ≈ 100 | 1 | 10-40 | 10-40 |
| Ncd236 | ≈ 1-100 | 10^-2^-1 | 5-20 | 5-2000 |
| Kif11(513) | ≈ 10 | 10^-1^ | 10-20 | 100-200 |
